# Prevalence of oral manifestations in children and adolescents with cancer submitted to chemotherapy

**DOI:** 10.1186/s12903-016-0300-2

**Published:** 2016-10-03

**Authors:** Deise Berger Velten, Eliana Zandonade, Maria Helena Monteiro de Barros Miotto

**Affiliations:** Federal University of Espírito Santo, Av. Mal. Campos, 1468-Maruípe, Victoria, 29043-900 Brazil

**Keywords:** Chemotherapy, Oral manifestations, Oncology

## Abstract

**Background:**

Oral complications may be observed during chemotherapy and are important side effects that may directly affect the anticancer treatment, even causing septicaemia in some cases. This research was done in order to evaluate changes in oral lesions during follow-up of children and adolescents in chemotherapy at Hospital Estadual Infantil Nossa Senhora da Glória (HEINSG).

**Methods:**

The study design was longitudinal, 45 patients were evaluated and monitored for 1 month after the initiation of chemotherapy. Twenty-eight patients were male and 17 female, ranging from 3 months to 18 years old.

**Results:**

The results show an increase in the number of mucositis cases and a decrease in xerostomia cases after the initiation of treatment, and other oral lesions were also found in low numbers.

**Conclusions:**

It is possible to avoid oral complications by maintaining a good oral health, and reducing infectious outbreaks. It is also feasible to obtain an early diagnosis of, and treat these oral complications, preventing them from following a more severe clinical course that may negatively affect the individual’s treatment. This outcome requires the presence of a dental surgeon on the multidisciplinary cancer treatment team.

## Background

Cancer is a term used to describe a group of diseases that involve the uncontrolled growth of cells [1]. In recent decades, cancer has become a serious global public health problem. The World Health Organisation (WHO) has estimated that in the year 2030, there will be 27 million incident cases of cancer, 17 million deaths from cancer, and 75 million people each year living with cancer. The regions most affected by these increases are developing countries [2].

Infant-juvenile cancers differ from adult cancers in many respects, such as types, sites, aetiologies, characteristics, and treatments [1].

Neoplasms were the leading cause (7 %) of deaths due to morbidity in children and adolescents (1–19 years) in 2011 and were surpassed by only deaths due to external causes. This fact is particularly troubling when one considers that younger individuals comprise a large proportion of the Brazilian population. According to information from the last population census in 2010, approximately 30 % of the population is below the age of 19 [3].

According to population-based cancer records (Registros de Câncer de Base Populacional), the percentage of paediatric tumours observed in Brazil is 3 %. Approximately 11,840 new cases of cancer in children and adolescents up to 19 years of age have been estimated in Brazil for 2014 [3].

There has been an improvement in the survival rates of children with cancer due to dosage intensification and the combination of chemotherapy drugs, which has also led to success in the cancer treatment healing response [4–6]. Chemotherapy acts by destroying or inhibiting the growth of rapidly multiplying cells, without differentiating cancer cells, which proliferate rapidly, from normal cells, such as the oral mucosa [7].

Chemotherapy is used to treat approximately 70 % of cancer patients [6, 8]. Of these patients, 40 % develop oral manifestations, and this number increases to over 90 % in children under 12 years of age [7, 9].

The following oral manifestations related to anticancer treatment are commonly found: mucositis, xerostomia [10, 11], infections, salivary gland dysfunction, dysgeusia, and pain [12].

Given that most infant-juvenile patients with malignancies receive chemotherapy, which is often associated with side effects consisting of oral complications, this study aimed to evaluate changes over the course of two monitoring sessions in the oral manifestations of children and adolescents receiving chemotherapy at the Hospital Estadual Infantil Nossa Senhora da Glória (HEINSG).

## Methods

This study had a longitudinal design and was performed by monitoring children and adolescents aged up to 18 years and 364 days who had received their first cancer diagnosis and had started chemotherapy at HEINSG between April 2013 and 2014. The individuals were evaluated on two occasions approximately 1 month after the initiation of chemotherapy.

When individuals were admitted to HEINSG and diagnosed with a malignant neoplasm, at the first study stage, an interview and clinical examination were performed before or within 3 days of starting chemotherapy. At this stage, data regarding oral health and sociodemographic characteristics, such as gender, age, place of birth, education, maternal education, and socioeconomic status, were collected.

The HEINSG serves its patients only by the Unified Health System (SUS) and it is located the only reference center of public state network for cancer treatment in children and adolescents. Being a public referral hospital usually the patients treated are less favored socioeconomic status.

The Economic Classification of individuals followed the New Economic Classification Standard Criteria of Brazil, which aims to discriminate large groups of people according to their consumption capacity of accessible products and services to a significant portion of the population and classify households, assuming, as an assumption that the class is a familiar feature among others. According to this criterion individuals can be classified into five classes A, B, C, D and E, and class A is the best economic status and class E the worst economic condition [13].

Some information, such as the indicated treatment and diagnosis, was collected from the patient’s medical records.

For information about xerostomia, mucositis, oral candidiasis, herpes simplex and cold sore, a clinical examination was conducted after completing the interview. All patients were examined in HEINSG dependencies by the same operator, which was a dentist (DBV), thus, diminishing the interobserver variation. A preparation was made through pictures of the most common oral alterations that would be expected to be found in the study group.

Through sialometry it is possible to evaluate the production of unstimulated saliva, or by chemical, mechanical or gustatory stimulation [14]. Individuals commonly produce 0.3 ml/min of saliva without stimulation; when secretion is less than or equal to 0.1 ml/min, it is characterised as hyposalivation [15].

An adaptation was made to the criteria proposed by the World Health Organization [16], a scale test that consists in measuring the production of saliva produced for one minute in a graph vessel (0 to 3 ml), to diagnose hyposalivation [14]. The test was performed after 1.5 to 2 h in which the patient should not have smoked, drunk, washed his mouth or swallowed food.

When the sialometry test performed in patients obtained a measure less than 0.3 ml/min. saliva, and moreover were present the symptoms described in any of degrees of severity scale used to quantify the xerostomia as a side effect (Table 1) proposed by the World Health Organization (WHO) xerostomia was considered present and was considered absent when the test there was a greater extent 0.3 ml/min and was also not observed any symptoms.

**Table 1:** Quantification of xerostomia as a side effect according to WHO

Degrees of severity of xerostomia	
Grade 1	Symptomatic (thick or sparse saliva) without significant dietary changes, saliva production unstimulated >0.2 ml/min.;
Grade 2	Symptomatic with significant changes in the oral intake (copious intake of water or use of other lubricants, limited to purees diet and/or soft and wet food); unstimulated saliva production from 0.1 to 0.2 ml/min.;
Grade 3	Symptoms leading to inability to feed orally; need for administration of intravenous fluids, enteral or parenteral nutrition; unstimulated saliva flow <0,1 ml/min.

Fonte. World Health Organization. Nacional Cancer Institute. Common Terminology Criteria for Adverse Events [16]

Xerostomia was only evaluated in children aged 6 years or more and in adolescents.

Mucositis was diagnosed via clinical examination by evaluating the appearance of the oral mucosa. Mucositis was not graded according to severity but was considered absent or present according to whether a change in the mucosa was noted on any of the severity scales (discoloration, erythema, pseudomembrane, or deep ulceration) proposed by the WHO [16].

Complications including oral candidiasis, labial herpes simplex, and mouth ulcers were evaluated by visual clinical examination [17].

### Statistical analysis

A descriptive analysis was performed on the sociodemographic characteristics and oral manifestations of the participants at the three study stages, according to their respective frequencies and percentages. The Kappa statistic was used to measure concordance in cases in which a particular individual presented an oral manifestation at the next stage.

McNemar’s test was used to verify changes in the number of cases of oral manifestations (direction of disagreement) between stages. A significance level of 5 % was adopted, and the software used in all analyses was IBM (SPSS) version 21.

All parents or guardians of the participants signed the consent form and previously clarified data collection. The study was approved on 20 February 2013 by the Research Ethics Committee of the Health Sciences Center (Health Sciences Center) of the Federal University of Espirito Santo (Federal University of Espirito Santo–UFES) by the number opinion 201,117.

## Results

**Fig. 1:**
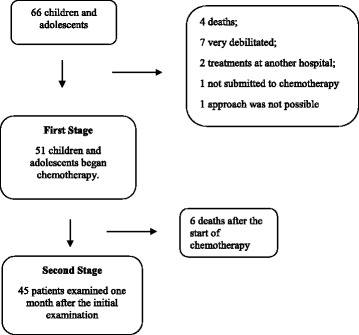
Deaths during the monitoring of patients at HEINSG

Between the months April 2013 and 2014, a total of 66 children and adolescents were admitted to HEINSG diagnosed with cancer to make chemotherapy. In 15 of these patients it was not possible to approach (Fig. 1). The initial study population was composed of 51 individuals so.

The final sample size was 45 children and adolescents at the second evaluation (1 month after the initial examination). It was not possible to follow six patients between the 1st and 2nd moments described in Fig. 1.

Table 2 shows the sociodemographic characteristics and diagnosis of the 45 individuals monitored during the study who were evaluated approximately 30 days after the initial examination, showing that most of the individuals were in the 1- to 4-year-old age group (31.1 %), were male (62.2 %), resided in greater Vitória (46.7 %), belonged to socioeconomic class C (62.2 %), attended school (73.3 %), and had up to 4 years of education (24.4 %). Regarding the children’s and adolescents’ families, the heads of most households were fathers (62.2 %). The education data clearly show that most household heads had up to 8 years of education (40 %), and most of the mothers had more than 12 years of education (35.5 %).

**Table 2:** Sociodemographic characteristics and diagnosis of 45 children and adolescents who were monitored in the study (2013 and 2014, HEINSG)

Category		Number	Percent
Age group	Less than 1 year	4	8.9
	1 to 4 years	14	31.1
	5 to 9 years	11	24.4
	10 to 14 years	6	13.3
	15 to 18 years	10	22.2
Gender	Male	28	62.2
	Female	17	37.8
City of residence/Home location	Greater Vitória–ES state	21	46.7
	City in ES state (other than the capital city)	18	40.0
	Bahia state	4	8.9
	Minas Gerais state	2	4.4
Socioeconomic class	B	7	15.6
	C	28	62.2
	D	9	20.0
	E	1	2.2
Head of household	Father	28	62.2
	Mother	3	6.7
	Grandfather/Grandmother	3	6.7
	Other	11	24.4
Education of household head (years of study)	0–4 years of study	4	8.9
	4–8 years of study	18	40.0
	8–12 years of study	12	26.7
	More than 12 years of study	11	24.5
Mother’s education (years of study)	0–4 years of study	3	6.6
	4–8 years of study	15	33.3
	8–12 years of study	10	22.2
	More than 12 years of study	16	35.5
	Not known	1	2.0
Diagnosis	Leukaemia	17	37.7
	Lymphomas	10	22.2
	Renal tumours	4	8.8
	CNS tumours	4	8.8
	Sympathetic NS tumours	3	6.6
	Bone tumours	2	4.4
	Other	5	11.1

Table 2 also shows that leukaemia was the most common diagnosis, occurring in 37.7 % of cases.

Table 3 shows the prevalence of oral complications found during the clinical examinations conducted during the two research stages. The prevalence of mucositis considerably increased between the first and second study stages, rising from 3.9 to 17.8 %. The prevalence of Xerostomia considerably decreased between the first and second stages, falling from 31 to 7.4 % of cases.

**Table 3:** Prevalence of oral manifestations at the two research stages

	Stages			
	1°		2°	
	*n*	%	*n*	%
Mucositis	2	3.9	8	17.8
Xerostomia	9	31	2	7.4
Cold sores	1	2	1	2.2
Candidiasis	2	3.9	4	8.9
Herpes simplex	0	0	1	2.2

Table 4 shows the changes in oral manifestations between the first and second study stages. A reduction in the number of cases of xerostomia was observed between the first and second stages, and the result of McNemar’s test was very close to the significance level, with a *p*-value of 0.06.

**Table 4:** Changes in oral manifestations between the first and second study stages

	Stage 1	Stage 2		*Kappa*	*P* value	McNemar *P* value
		Yes	No			
Mucositis	Yes	0	2	−0.077	0.999	0.109
	No	8	35			
Xerostomia	Yes	2	5	0.369	0.065	0.063
	No	0	19			
Cold sores	Yes	0	1	−0.023	0.999	0.999
	No	1	43			
Candidiasis	Yes	2	0	0.646	0.006	0.500
	No	2	41			

## Discussion

Approximately 40 % of adult cancer patients and more than 90 % of children under 12 years of age undergoing cancer treatment have oral manifestations that directly or indirectly arise from stomatotoxicities, such as mucositis; xerostomia; fungal infections, such as candidiasis; and viral infections, such as labial herpes simplex [7, 9].

Many national and international studies in the literature show a correlation between anticancer treatments, particularly chemotherapy, and the occurrence of oral complications in infant-juvenile patients and adults [8, 11, 18–27], and the magnitude of these side effects depends on a number of factors related to the treatment, tumour, and patient [23].

A less favored socioeconomic status is usually related to a greater number of oral diseases such as caries, i.e., a more unfavorable oral condition, which increases the risk of oral manifestations during chemotherapy [34]. In this study, however this association was not proven statistically, probably because patients studied belonged mostly to classes B, C and D, with a greater concentration in class C, which made it difficult to compare the classes. Our results therefore showed that oral manifestations were not related to the socioeconomic status of patients.

Oral mucositis is defined as an inflammation and ulceration of the oral mucosa [28–32] and is one of the most common and frequent lesions in cancer patients undergoing chemotherapy. It is a major cause of pain and also represents a distressing experience for cancer patients [33, 34].

In this study, an increase in oral mucositis cases was observed between the first and second stages. In addition, mucositis was the most common oral manifestation found in the clinical examination conducted 1 month after the beginning of chemotherapy. This result is similar to those found in other studies [23, 24, 27, 35] conducted in Brazil (Recife, Juiz de Fora, São José dos Campos, and João Pessoa) and abroad (China). Other authors [26] have observed mucositis percentages higher than those found in this study. The results of research conducted in São Luis (Brazil) [18] were different than those of this study, as few cases of mucositis occurred during anticancer treatment for leukaemia. This finding was likely due to the small sample, which included only 12 individuals. The literature varies regarding the prevalence of oral mucositis induced by chemotherapy, which has been reported to be 40 % [22], 65 % [33] and between 52 and 81 % [33]. However, in general, the results reported in the literature have been higher than those found in this study, which is likely due to favourable oral health conditions before the initiation of treatment, with a low percentage of oral diseases, which reduces the risk of developing oral manifestations during treatment [7]. In this study, children and adolescents with various types of cancer were examined, which may, to some extent, be responsible for the low prevalence of mucositis, whereas in studies concentrating solely on leukaemia patients, generally speaking, a higher prevalence of oral manifestations was encountered because leukaemia is characterised by a high incidence of oral lesions during treatment [35].

Tests used in this study for measuring salivary flow were an adaptation of the standards proposed by the World Health Organization for quantification of xerostomia as a side effect of chemotherapy. Usually, authors recommend saliva collection for 5 min to measure the flow, but in adults [14]. In the present study, patients evaluated were children and adolescents who showed a severe general condition, sometimes incapacitating, so we chose to perform this adaptation in sialometry test, reducing saliva collection time.

In this study, the number of xerostomia cases declined between the first and second stages. Different results were found by other estudos [11, 24] of leukaemic patients [27] in which there was an increase in the number of cases of xerostomia between stages. It is likely that in this study, the reduction in xerostomia cases after the start of chemotherapy (which was close to the significance level, reaching 6 %) was due to the care given during treatment, as an increased number of cases of leukaemia can be observed among patients, and the majority of individuals with leukaemia remained hospitalised, received continuous intravenous hydration, and also followed guidelines regarding the need for extensive water and liquid intake in the first month of treatment.

A low prevalence of oral candidiasis was found in this study, which is consistent with other studies [24] and differs from the results obtained in one study [27] of leukaemia patients. The low prevalence of oral candidiasis (a result of the myelosuppression caused by chemotherapy agents) was likely due to the use of antifungal drugs that act topically and also systemic during the treatment [35] of most patients. Antifungal medicaments used were fluconazole 100 mg, once a day and nystatin, three times daily. These medicaments were prescribed by the hospital crew at the first signs of oral candidiasis, perhaps this influenced the low prevalence of this disease. These antifungals are not known to cause salivary flow reduction.

Patients with better oral health conditions and satisfactory oral hygiene develop fewer oral manifestations, and those conditions that do occur have a more rapid clinical course [35]. Studies have reinforced the importance of oral health for the prevention and reduction of oral complications of cancer treatment [10, 31], so prior to chemotherapy it is important to perform a Stomatological evaluation and dental care to reduce infectious outbreaks, such as removal of carious lesions and extensive restorations, treatment of periodontal disease and even tooth extractions in cases of teeth that require prolonged treatment, as all infectious processes originated in the oral cavity present a high risk of systemic infection, which can lead to septic episodes in immunocompetent patients compromised by oncologic therapy.

The presence of a dental surgeon working directly on the medical team is essential for the prevention and treatment of oral manifestations resulting from antineoplastic treatment. This dental surgeon can assist with preventing the development of a more severe clinical course, which can even result in the suspension of antineoplastic treatment [24, 35, 36].

The sample size was a limitation of this study. However, in Brazil, the observed percentage of paediatric tumours is 3 %, demonstrating the low prevalence of this disease, which makes allocating a large number of individuals difficult in a primary data study.

## Conclusions

Based on the results observed in this study, it can be concluded that oral manifestations were present, including mucositis, which increased, and xerostomia, which showed a reduction in the number of cases after the start of chemotherapy. Oral complications, despite their relatively low prevalence, were nonetheless present and are among the most devastating complications in both the short and long term, affecting basic human activities such as eating and communicating. Furthermore, these lesions may interfere with cancer treatment, causing more serious infections or even sepsis.

The presence of an active dental surgeon on the multidisciplinary oncology team is therefore indispensable, as they can assist in the prevention, early diagnosis, and treatment of oral manifestations. A dental surgeon can prevent lesions from escalating, thereby improving the patient’s quality of life during treatment.
